# Protein-Protein Interactions in Clathrin Vesicular Assembly: Radial Distribution of Evolutionary Constraints in Interfaces

**DOI:** 10.1371/journal.pone.0031445

**Published:** 2012-02-22

**Authors:** Rupali A. Gadkari, Narayanaswamy Srinivasan

**Affiliations:** Molecular Biophysics Unit, Indian Institute of Science, Bangalore, India; University College Dublin, Ireland

## Abstract

In eukaryotic organisms clathrin-coated vesicles are instrumental in the processes of endocytosis as well as intracellular protein trafficking. Hence, it is important to understand how these vesicles have evolved across eukaryotes, to carry cargo molecules of varied shapes and sizes. The intricate nature and functional diversity of the vesicles are maintained by numerous interacting protein partners of the vesicle system. However, to delineate functionally important residues participating in protein-protein interactions of the assembly is a daunting task as there are no high-resolution structures of the intact assembly available. The two cryoEM structures closely representing intact assembly were determined at very low resolution and provide positions of Cα atoms alone. In the present study, using the method developed by us earlier, we predict the protein-protein interface residues in clathrin assembly, taking guidance from the available low-resolution structures. The conservation status of these interfaces when investigated across eukaryotes, revealed a radial distribution of evolutionary constraints, i.e., if the members of the clathrin vesicular assembly can be imagined to be arranged in spherical manner, the cargo being at the center and clathrins being at the periphery, the detailed phylogenetic analysis of these members of the assembly indicated high-residue variation in the members of the assembly closer to the cargo while high conservation was noted in clathrins and in other proteins at the periphery of the vesicle. This points to the strategy adopted by the nature to package diverse proteins but transport them through a highly conserved mechanism.

## Introduction

Intracellular transport of biomolecules is an important event for the functioning of a cell. Both, endocytic as well as exocytic pathways of trafficking in eukaryotic cells involve formation of caged vesicles that communicate between the organelles of the same cell or to the exterior of the cell [1]. Clathrin coated vesicle system (CCVs) is responsible for receptor-mediated endocytosis at the plasma membrane besides sorting of proteins at trans-Golgi during biogenesis of lysosomes and secretory granules [2]. In the recent times, diverse nature of the functions carried out by CCVs is becoming evident [3]. These vesicles have been implicated in spindle organization and stabilization during both mitosis [4] as well as meiosis [5]. Thus CCVs actively participate in chromosome segregation during cell divisions and this function is independent of its function of endocytosis. Also, this assembly actively participates in Golgi reassembly post mitosis. Owing to the functional importance of this assembly in eukaryotic organisms, these vesicles have been subject of intense research in the past several decades [6], [7], [8], [9].

**Figure 1:**
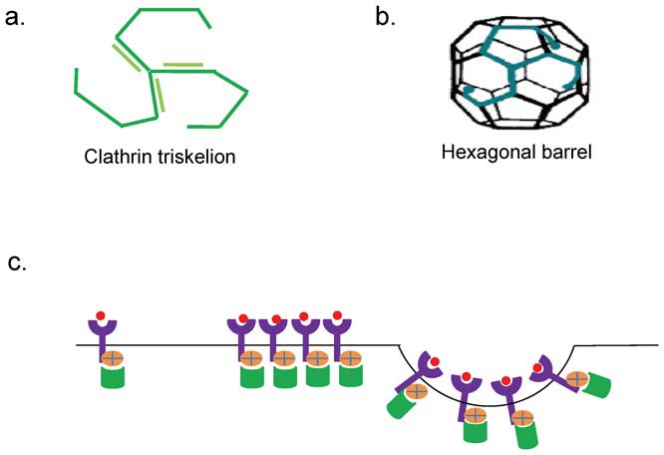
Components of Clathrin coated vesicles. a] The basic functional unit of clathrin cage is clathrin triskelion. The triskelion consists of three clathrin heavy chains (dark green) interacting non-covalently with clathrin light chains (shown in light green). b] The clathrin chains surrounding the cargo polymerize to form a hexagonal barrel inside which the cargo is transported from one place to another safely. c] When the cargo is to be transported from one place to another it starts getting accumulated at the membrane (cargo shown as red spheres & membrane as black horizontal line), bound to its receptor (shown in purple). The cargo receptors recruit adaptor proteins (the heterotetramers in orange), which in turn employ clathins (in green). With the help of accessory proteins recruited by clathrins the plasma membrane invaginates and the clathrin coated vesicle is clipped off subsequently.

Clathrin, a cytosolic protein, was identified as the major component of CCVs and hence the name [7]. The basic functional unit of clathrin is a clathrin triskelion (Figure 1a), which consists of three clathrin legs interacting to form a vertex [10], [11]. Clathrin leg comprises of a heavy chain interacting non-covalently with a light chain. Under mild acidic conditions clathrins can spontaneously polymerize to form a basket-like protective compartment to ferry proteins, as shown in the Figure 1b [12] [13]. The components of clathrin coated vesicular assembly can be broadly grouped into three layers. The inner membrane layer embeds the cargo and is linked to the outer clathrin lattice by a layer of cargo-binding adaptor proteins that aid and regulate vesicle formation (Figure 1c). Depending upon the site of activity various adaptor as well as accessory proteins are recruited in the vesicle to carry the specific cargo [14].

Protein-protein interactions play a crucial role in maintaining the structural integrity and functional state of the assembly [15]. In an intact, functional clathrin coated vesicle there are close to 400 polypeptide chains that interact closely [16]. Often, a polypeptide chain interacts with more than one protein partner [17]. Clathrin chains are the permanent members of the assembly while the other components are recruited on the need basis and vary depending upon function to be carried out by CCVs [18]. The components of CCV can be classified into three categories namely clathrins, adaptor proteins or adaptins and accessory proteins apart from the cargo and its receptor, as can be seen in the Figure 1c. Adaptins, the heterotetrameric protein complexes [19], are the busiest members of the assembly in terms of the protein partners that they engage and hence, are often termed as hubs of this interaction network [20]. In mammalian cells there are four such adaptin complexes designated AP1 through AP4, each of which works in a particular signaling cascade [21]. Such adaptor proteins possess a trunk domain that interacts with cargo and lipid layer and two appendage domains on flexible linkers which interact with the accessory proteins as well as clathrin heavy chains [22], [23]. The next busy component or second hub of the assembly is formed by clathrin chains. The clathrin heavy chains consist of three domains; a terminal domain, a distal domain forming knee of the clathrin leg and a proximal domain that is closer to C-terminus forming ankle region. The terminal domain interacts with adaptins and accessory proteins forming yet another hub in the clathrin assembly network [24], [25]. The distal domain that possesses CHC repeats provides strength to the clathrin lattice by interacting with similar domains from other clathrin heavy chains in the vicinity while the proximal domain is engaged in holding the triskelion structure by interacting with other two heavy chain proximal domains [2]. Depending upon the function to be carried out by the CCVs the accessory and adaptor proteins in the assembly change while clathrin heavy and light chains are maintained the same. Hence, it is important to understand how these interactions are orchestrated. A number of relevant questions such as “Through which regions do these proteins interact specifically with their multiple partners?”, and “How comparable are these interactions, in terms of residue contributions, across different eukaryotic species?”, have remained unanswered as there are no high enough resolution structures of the intact clathrin vesicular assembly. The only insightful structures those are available are the two cryo-electron microscopic structures namely of clathrin coats with and without part of one of the accessory proteins, auxilin [26], [27]. These structures provide excellent insights on the overall structure of the outer coat of the assembly. However, deriving residue level structural information is a rather difficult task as these structures have been solved at very low resolutions, which permit elucidation of the structure only at the level of Cα atoms.

In the present analysis, we have made use of these low resolution cryo-EM fitted models to gain better insights onto the protein-protein interactions made by clathrin chains. Towards this, we have used the method developed by us earlier, that can predict protein-protein interactions interface residues with high sensitivity and accuracy, starting from low resolution structures providing Cα atom positions only [28]. The method uses solvent accessibility criterion to adjudge the propensity of a residue to participate in protein-protein interactions and was successfully used earlier to elucidate the changing interaction interfaces in dengue virus coat protein E and M, from low resolution cryoEM structures, during the process of maturation of the virus [29]. Application of the method to clathrin coated assembly structures enabled us to predict the functionally relevant regions in the clathrins and this prediction is strongly anchored on the basis of low resolution cryoEM derived structures. To gain better understanding of the communication between the components of the CCVs, the appropriate structures involving other components were analyzed and residues participating in interactions were dissected out. The conservation status of the interaction interfaces across eukaryotes was investigated subsequently.

## Methods

### Structures analyzed

Table 1 provides a comprehensive list of the structures of CCV components used in the present analysis along with the resolution at which the structures were solved. The structural data was obtained from RCSB protein data bank [30].

**Table 1:** Structures of the components of clathrin coated assembly analyzed.

PDB ID	Description	Resolution	Organism
*Clathrins*			
1xi4	Clathrin D6 coat	8 Å	bovine
1xi5	Clathrin coat with auxillin J domain	8 Å	bovine
1b89	Clathrin heavy chain proximal leg	2.6 Å	Bovine
1c9i	Beta propeller with peptides	2.9 Å	rat
1bpo	Heavy chain terminal domain	2.6 Å	rat
1utc	Terminal domain with amphiphysin peptide	2.3 Å	Bovine
1c9l	Beta-propeller with peptide	2.9	Rat
*Adaptor proteins*			
1ky6	AP2 α-appendage with Epsin peptide	2 Å	Mouse
1ky7	AP2 α-appendage with amph peptide	2.1 Å	Mouse
1kyu	AP2 α-appendage with EPS15 DPF pep	1.8 Å	Mouse
1kyd	AP2 α-appendage with Epsin peptide	2 Å	Mouse
1kyf	AP2 α-appendage with EPS15 DPF pep	1.2 Å	Mouse
1qts	AP-2 adaptor α-appendage	1.4 Å	Mouse
1qtp	AP-2 adaptor α-appendage	1.6	Mouse
1gyu	AP-1 adaptor γ-appendage	1.81	Mouse
1gyv	AP-1 adaptor γ-appendage mutant	1.71	Mouse
2vj0	AP2 α-appendage in complex with FXDNF from amph & WVXF from synaptojanin	1.6	Mouse & synthetic
1gyw	AP1 γ-appendage A753D mutant	2.4	Mouse
2iv8	β1 of AP2 with β-arrestin peptide	2.8	Human
1w63	AP1 adaptor core	4	Mouse & rat
1w80	α-adaptin of Ap2 with 2 peptides from synaptojanin 170	1.9	Mouse & synthetic
2g30	β-appendage of AP2 with ARH peptide	1.6	Human & synthetic
2ivg	β-appendage of AP2 with EPS15 peptide	1.9	Human
2vgl	AP-2 adaptor core	2.6	Rat, human & mouse
1e42	β2 of AP2	1.7	Human
1b9k	α of AP2	1.9	Mouse

Listed are the cryo-EM fitted models or the structures of the components of CCV determined using X-ray crystallography.

### Recognition of protein-protein interaction interfaces

Protein-protein interaction interfaces of the components of CCV were recognized using accessibility criterion. As can be seen in the Table 1, some of the structures have been determined at very low resolutions and they provide Cα atom positions only. In such cases the new method developed in house was used to recognize protein-protein interaction interfaces [28]. Briefly, our method mimics the classical approach used for protein-protein complex structures with all the atomic positions available and using the solvent accessibility calculations [31]. The accessible surface area values in the low resolution structures have been calculated using a spherical probe with larger radius of 3.5 Å while in case of high resolution structures with all atom positions probe of 1.4 Å radius was used. In the high resolution structures a residue is said to be present in the interaction interface if it is buried in the complex form (Accessibility<7%) and exposed in the isolated form (Accessibility>10%) [32]. In our method we have defined the residue type-dependent cutoff values for accessible surface area values of Cα atoms that are corresponding to 7% and 10% accessibility values. Using these limits the residues participating in the protein-protein interactions were identified. For the structures solved at higher resolution interfaces were identified using standard limits of accessibility values mentioned above.

### Conservation status of interfaces

The homologues of human clathrin chains as well as adaptins were identified across eukaryotic organisms by carrying out sequence search using PSI-BLAST [33] against all the eukaryotic genomic data available till date. The sequences showing greater that 30% sequence identity with the query sequence and covering greater than 70% of query sequence length were selected for further analysis. Subsequently multiple sequence alignments were carried out amongst the selected sequences using ClustalW [34]. To investigate conservation of the interface residues in the above mentioned multiple sequence alignments (MSA), a popularly used software “Consurf” was used [35]; [36]. When MSA is provided as an input to the software it computes a conservation score which is a relative measure of evolutionary conservation at each sequence site of the target chain. The lowest score thus, represents the most conserved position in a protein. It does not necessarily indicate 100% conservation (e.g. no mutations at all), but rather indicates that this position is the most conserved in this specific protein calculated using a specific MSA. Using this method the conservation scores were calculated for every position in every subunit of clathrin and adaptins.

### Phylogenetic analysis

1] Tree construction- Using the multiple sequence alignments mentioned above, the phylogenetic trees were constructed for clathrin chains as well as the components of the adaptor protein complexes. The tree constructions were carried out using PHYML programme [37] [38] that builds the phylogenetic trees using maximum likelihood approach, using the default parameters. The model of evolution was assumed to be based on the LG model [39], that utilizes the capability of maximum likelihood estimation and incorporates the rate heterogeneity concept at different sites in the construction of the amino acid substitution matrix.

2] Correlation of genetic distances- Using the trees constructed as mentioned above and the multiple sequence alignments mentioned previously, the genetic distance matrices of n×n orthologous sequences was computed using TREE-PUZZLE [40]. The similarity between genetic distance matrices of a pair of interacting proteins (or non-interacting proteins) was calculated using standard Pearson's correlation co-efficient. To assess the significance of the correlation coefficient, the observed correlation coefficient values were evaluated against values from unrelated protein components of the assembly namely between the functionally non-equivalent chains of two different adaptor protein complexes.

## Results

### Structural information about CCV components

1. **Structure of clathrin cage.** As mentioned earlier, there is no structure available for the intact assembly of clathrin coated vesicles. The structures that resemble the overall assembly closely are the two cryo-EM structures of empty clathrin cage, with or without part of auxilin J domain [26]; [27]. However, these structures are available at very low resolutions (12 Å and 8 Å respectively) and are available only at the level of positions of Cα atoms. These models were generated by superimposing on the cryo-EM density maps the high resolution structural data of clathrin chains namely that of clathrin triskelion (PDB ID: 1bpo) [41] and of the proximal leg of clathrin heavy chain (PDB ID 1b89) [42].
2. **Adaptor proteins.** Out of the four different types of adaptor proteins structural information is available for only two complexes namely adaptor protein 1 (AP1, PDB code 1w63) [21] and adaptor protein 2 (AP2, PDB code 2vgl) [43]. These structures reveal the molecular details of the cores of these complexes while separate structures provide the information about the appendage domains bound to their non-adaptin partners namely clathrin heavy chain and one of the accessory proteins epsin etc. (PDB ID 1c9i and 1kyd respectively); [44] [45]. Besides one structure (PDb code 2xa7) of the adaptor protein AP2 in complex with the cargo receptor is available in the protein data bank [46].

Apart from the above mentioned structures that were used in the main analysis, a number of other structures were used as supporting structures to confirm our predictions. The complete list of the structures analyzed is given in the Table 1.

### Protein-protein interaction interfaces of clathrin cage

**Figure 2:**
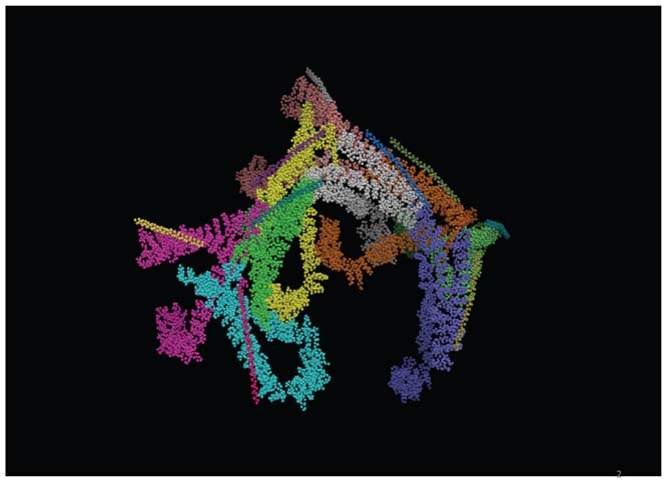
Structure of Clathrin coat (PDB ID : 1xi4). Shown in the figure is the structure of clathrin coat, visualized in 3D using PyMOL software [53]. The structural model was generated by superimposing high resolution structural data over the low resolution cryoEM electron density by Fotin A and coworkers [26]. The model was provided at a resolution equivalent to 8 Å and it provides Cα atom positions only. Shown in the figure are the clathrin chains with the Cα atoms represented as spheres. The light chains of clathrin are seen as slender sticks in the figure while others occupying most of the space are the heavy chains.

**Figure 3:**
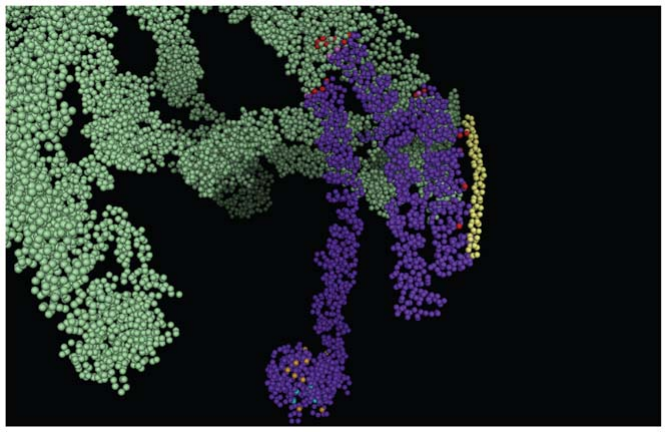
Interface residues of clathrin heavy chain. The figure provides closer view of one of the heavy chains in the structure of clathrin coat (shown in figure 2) and its residues interacting with various components of the vesicular assembly. The clathrin heavy chain is shown in purple and clathrin light chain in yellow. The red spheres depict the residues of heavy chain interacting with other clathrin chains (either light chains or other heavy chains), pink spheres are the residues in interaction with auxillin peptide (an accessory protein) while orange spheres are the residues interacting with adaptor protein chain and the residues forming interface with amphiphysin peptide (another example of accessory protein) are in cyan.

Recognition of protein-protein interaction interface in case of clathrin cage was a twofold problem; a] The structures available for the clathrin cage provide positions of only Cα atoms and hence recognition of interface was a non-trivial task and b] To further add to the complexity, the structural models comprise eighteen polypeptide chains (as shown in the Figure 2) and hence, were difficult to process for computing solvent accessible surface area of every residue. We have developed a method which can recognize the protein-protein interaction interfaces solely from Cα positions in low resolution structures of big assemblies such as CCV [28]. However, prior to applying this method, in order to circumvent the second problem mentioned above, we identified near neighbors for every chain in the complex structures using distance criterion; if the distance between two Cα residues from different chains is less than or equal to 5 Å then the chains possessing the residues are termed as near neighbors. The complex structures (PDb IDs 1xi4 & 1xi5) were then divided into smaller sub-complexes that were treated as independent structures to recognize interface residues. These sub-complexes are listed in the Table 2. Subsequently, the interaction interfaces were recognized using the protocol as described in Methods section and are listed in the Table 3 and Table 4. As can be clearly seen in the tables, all the heavy chains in the structures contribute differently although there is an overlap in terms of the interacting residues. The Figure 3 shows interface residues recognized in case of G chain of 1xi4 and as is clear from the picture, our method has indeed identified the interface residues specifically from Cα positions available. When the interface predicted in clathrin coat was compared with that of clathrin coat with auxilin peptide bound to it, it was clearly seen that auxilin chains were bound to the terminal domain of clathrin heavy chain (Figure 3) (Table 4). Thus, it clearly reconfirmed the known fact that terminal domain of clathrin interacts with other non-clathrin components while the interactions between clathrin chains are restricted to the leg region of the chain [26], [27].

**Table 2:** Subcomplexes of clathrin coat structures.

Chain	Chain in Complex for 1xi4	Chain in Complex for 1xi5
A	AEFJB	ACFN
B	BCDK	BCD
C	CDLB	CDA
D	DCEMF	DECB
E	EADHNF	EDH
F	FADEGO	FAGHR
G	GFPIH	GHIF
H	HEGQ	HEFM
I	IGHR	IGH
J	JA	-
K	KB	-
L	LC	-
M	MD	MHEF
N	NE	NACF
O	OF	-
P	PG	-
Q	QH	-
R	RI	RFAGH

Clathrin coat structures (with and without auxillin; 1xi5 and 1xi4 respectively) were dissected into smaller subcomplexes by identifying near neighbors of the chains that are designated in the structures by letters A to I for clathrin heavy chains and J to R for clathrin light chains.

**Table 3:** Interface residues of clathrin heavy chains as predicted from low resolution structure of clathrin coat (PDB Id 1xi4).

Chain A	Chain B	Chain C	Chain D	Chain E	Chain F	Chain G	Chain H	Chain I
L845	R444	L820	T842	G443	L845	L820	T842	A1355
V849	A1355	E826	L845	R444	E848	K830	L845	F1414
R852	E1475	K830	E848	L820	V849	I833	E848	E1475
R854	L1504	I833	V849	E826	R852	R837	V849	L1504
W861	E1584	R837	R852	K830	R854	N1248	R852	E1584
I866	W1587	N1248	R854	I833	W861	F1258	R854	W1587
H867	D1614	F1258	W861	R837	E863	V1261	W861	I1591
E868		V1261	E868	N1248	E868	F1266	E868	D1611
E896		Q1270	E1282	V1261	E896	Q1270	L1283	D1614
L1283		L1274	L1283	Q1270	V1277	G1273	L1286	S1618
Y1290		I1276	L1286	G1273	E1282	L1274	Y1290	
M1302		V1277	Y1290	L1274	L1283	I1276	M1302	
A1306		V1278	M1302	I1276	L1286	V1277	A1306	
L1309		H1279	A1306	V1277	Y1290	V1278	A1355	
R1311		A1355		V1278	M1302	H1279	F1414	
A1355		E1475		H1279	A1306	A1355	E1475	
E1475		L1504		A1355	F1414	F1414	L1504	
L1504		E1605		F1414	E1475	E1475	M1596	
E1584				E1475	L1504	L1504		
W1587				L1504		E1584		
				E1584		W1587		
				W1587		D1611		

**Table 4:** Interface residues of clathrin heavy chains as predicted from low resolution structure of clathrin coat with axillin peptides bound to the heavy chains (PDB Id 1xi5).

Chain A	Chain B	Chain C	Chain D	Chain E	Chain G	Chain H	Chain I
R8	P408	P813	V341	F762	E703	R8	D1580
L13	E1584	G817	V849	L820	F762	Q10	E1584
E330	D1614	L820	L857	E826	G817	L13	W1587
F762		V822	P860	N1248	L820	N17	I1591
V849		D823	W861	V1261	D821	S326	M1603
R854		S825	E868	Q1270	E826	L845	L1607
L857		E826	L1268	G1273	T1250	V849	V1610
W861		I833	M1271	I1276	K1254	R854	D1611
L1283		T1250	L1283	V1277	F1258	E868	D1614
L1286		K1254	L1286	N1420	M1271	K951	S1618
Y1290		F1258	Y1290	M1424	G1273	L1013	
E1298		G1273	E1298	Y1598	L1274	L1283	
M1302		L1274	M1302	F1599	I1276	L1286	
A1306		I1276	D1580	Q1601	V1277	Y1290	
Y1598		V1277	E1584	E1605	V1278	M1302	
F1599		V1278	W1587	D1611	H1279	T1396	
Q1601		H1279	I1591		E1584	M1424	
E1605		D1580	L1607		W1587	V1425	
D1611		E1584			Y1598	S1427	
		W1587			F1599		
		I1591			Q1601		
		M1603			E1605		
		L1607			T1608		
		Q1630			D1611		
					D1614		
					S1618		

### Protein-protein interaction interface of adaptor proteins

Adaptor core: As mentioned earlier the adaptor protein complex consists of four different chains (AP1 has chains α, β1, σ and μ while AP2 has γ, β2, σ and μ). Using the standard method harboring accessibility criterion the residues of adaptor protein subunits involved in protein-protein interactions were recognized. Two structures available of the cores of adaptor protein AP1 (PDB code 1w63) [21] and AP2 (PDB code 2vgl) [43] were analyzed for this purpose. In the case of structure of AP1; 1w63; the complex structure was divided into subcomplexes to overcome the constraint imposed by its bulk. Apart from the above mentioned two structures there is a structure of adaptor protein in complex with the cargo receptor peptide [46]. By analyzing the interaction interfaces in this structure we could identify the interface region on the μ subunit (chain M in structure) that is involved in interaction with the cargo receptor, which is distinct from its interface with the core of the adaptor protein complex. The table 5 shows the comparison between the interfaces identified for the μ subunit in the two different structures as mentioned above. The region in interface with cargo receptor is shown in red in the table. This region harbours T156 residue which is known to get phosphorylated, which increases the receptor binding affinity of the subunit [3].

**Table 5:** Interface residues of the μ subunit (M chain in structure) of the adaptor protein AP2 from core structure (PDB code: 2vgl) and the core structure bound to cargo receptor peptide (PDB code: 2xa7).

Interface residues in core structure (PDB 2vgl)	Interface residues in core structure bound to cargo receptor peptide (PDb 2xa7)
PRO 46	PRO 46 M
VAL 47	VAL 47 M
SER 54	SER 54 M
ALA 75	VAL 58 M
ALA 76	ALA 75 M
MET 77	ALA 76 M
PHE 79	MET 77 M
TYR 109	PHE 79 M
GLU 110	TYR 109 M
GLU 114	PHE 118 M
PHE 118	TYR 120 M
TYR 120	PRO 121 M
PRO 121	GLN 122 M
GLN 122	SER 124 M
SER 124	ILE 151 M
SER 186	THR 152 M
LEU 192	VAL 155 M
ILE 241	THR 156 M
ILE 290	LEU 184 M
VAL 306	ASP 256 M
LYS 420	THR 258 M
VAL 422	LYS 431 M
	VAL 433 M
	GLU 443 M

**Figure 4:**
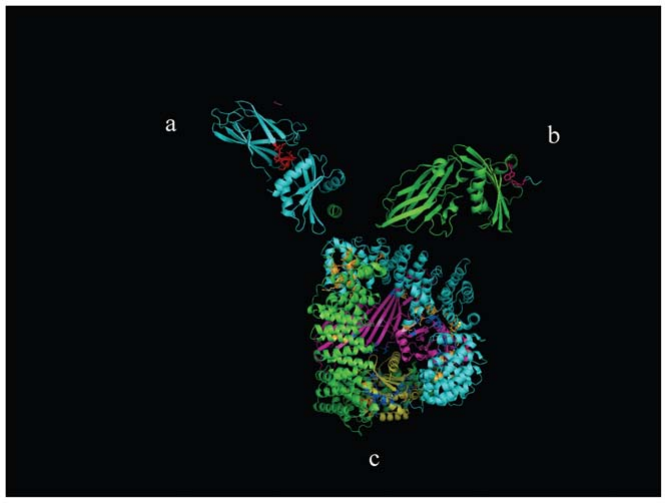
Structure of adaptor protein 2. The figure is a collage of three different structures available of the components of AP2 complex, generated, using PyMOL software [53], to provide an overall view of the entire AP2 complex. a] Structure of appendage domain of B chain (in cyan) with interface residues interacting with clathrin heavy chain peptide (shown in red); Towards this the interface residues on AP2 chain B in PDB structure 1c9i were mapped on to and highlighted in the structure of entire appendage domain (PDB id 2vi8). b] Structure of appendage domain of A chain of AP2 (shown in green) with the residues interacting with one of the accessory proteins arrestin shown in pink (PDB id. 1kyd). c] Structure of core AP2 (PDB id. 2vgl) with B chain in cyan, A chain in green, M chain in magenta and S chain in yellow while the residues in the interactions with the other chains in the structure are highlighted in either orange or blue.

The above mentioned structures lacked the appendage region in the beta chains of both the adaptins. This gap in the information was filled by analyzing the high resolution structures of these regions namely the PDB ids: 2iv8 [47], 1kyd [45] and 1c9i [44]of AP2. In these structures the appendage domains are in association with different accessory proteins, namely AP2 α appendage with epsin in case of 1kyd while AP2 β-appendage with beta-arrestin in case of 2iv8 and with CHC terminal domain structure. The information acquired in bits and pieces was then collated to obtain overall picture of the regions on various subunits of adaptins participating in different interactions as summarized in the Figure 4.

### Conservation of interface residues

**Figure 5:**
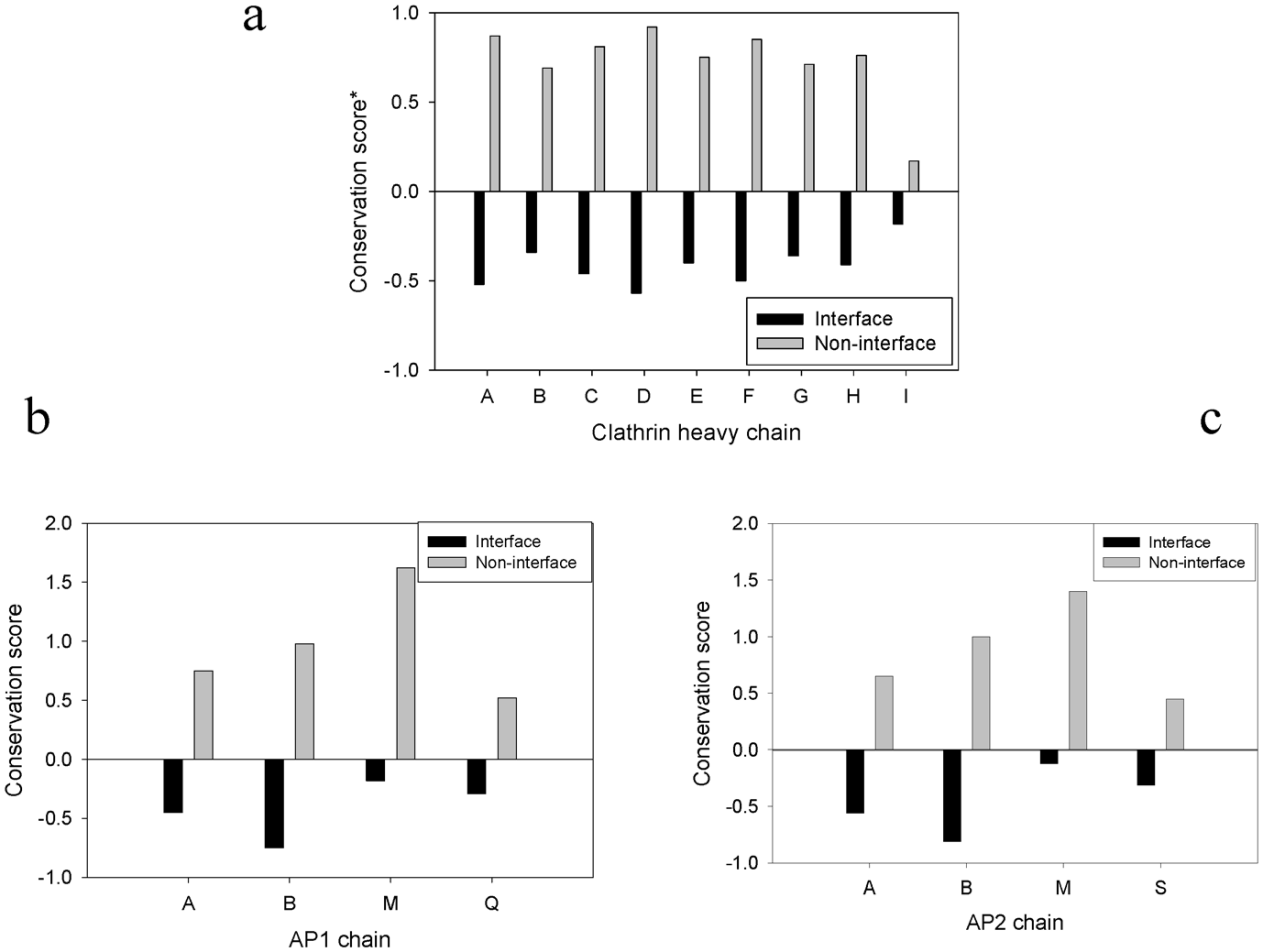
Conservation status of interface residues of clathrin vesicle assembly components. Residue conservation scores were calculated using Consurf (as described in “Methods” section). The relative measure of the evolutionary conservation at every position in the subunit was averaged for the interface residues and non interface surface exposed residues. The figure provides comparative picture of the conservation scores for the interface residues and non-interface surface exposed residues of clathrin heavy chains (shown in “a” panel), chains of adaptor protein 1 complex (b panel) and the chains of adaptor protein 2 complex (shown in “c” panel).

In a given polypeptide chain the residues participating in protein-protein interactions are often conserved better over the course of evolution compared to their non-interface solvent exposed regions. The residues identified as interface residues in case of clathrin chains when tested for residue conservation were also found to be better conserved compared to the non-interface, surface exposed residues of the same chain as shown in the Figure 5a. To analyze this aspect more quantitatively, the software Consurf was used to calculate conservation scores for every position in every subunit of clathrin and its adaptor proteins in the assembly. Figure 5a shows the comparison of conservation scores for predicted interface residues and non-interface surface exposed residues of clathrin heavy chains of the clathrin coat structure (PDB ID 1xi4) while that in case of adaptins is shown in the Figure 5b (for AP1 chains) and 5c (for Ap2 chains).

When conservation of interface residues were compared between different components of the assembly it was observed that the interfaces were maximally conserved in clathrin heavy chain with B chains of adaptor proteins ranking next. Minimum residue conservation was observed in the interfaces of the chains of the adaptor proteins that directly interact with the cargo receptors (μ chains of both the adaptor protein complexes). To investigate this observed pattern further and to attain a quantitative picture, a detailed analysis of evolutionary constraints over these protein chains was carried out subsequently.

### Phylogenetic analysis

**Figure 6:**
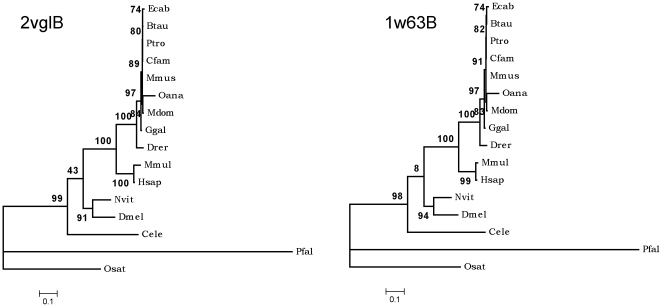
Phylogenetic tree topology comparison-1. The figure provides comparative picture of phylogenetic trees of the functionally quivalent chains of the two adaptor protein complexes namely AP2B (2vglB) and AP1B (1w63B). The phylogenetic trees were constructed using PHYML programme, using maximum likelihood method (as described in Methods section).

**Figure 7:**
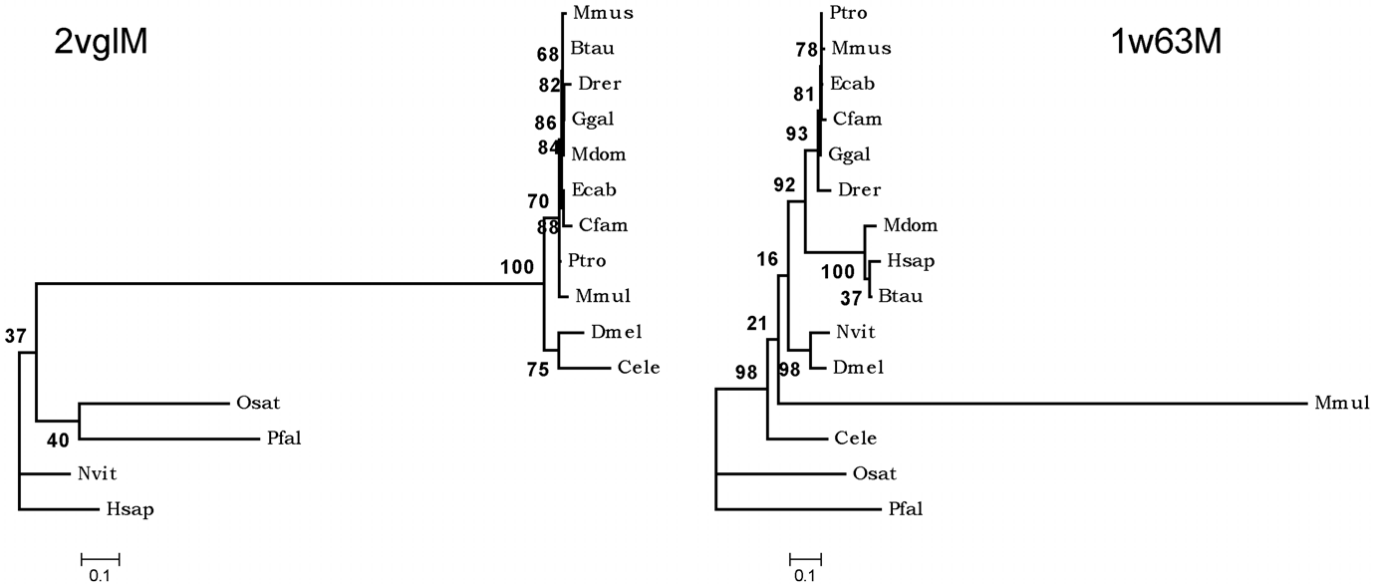
Phylogenetic tree topology comparison-2. Comparative picture of phylogenetic trees between functionally equivalent pair of subunits from the two adaptor protein complexes, AP2M (2vglM) and AP1M (1w63M). The trees were constructed as mentioned in the legend to Figure 6 and in the merthods section.

Using the multiple sequence alignments obtained using ClustalW, phylogenetic trees were constructed using PHYML, which constructs maximum likelihood tree based on the alignment. Comparative analysis of the constructed phylogenetic trees unfolded some of the interesting facets of the evolutionary divergence pattern amongst the subunits of the two prominent hubs of the clathrin coated vesicle assembly namely clathrins and adaptor protein complexes. The orthologous sequences that were compared were taken from the identical set of organisms. The key observations of the analysis were as follows;

1] When the functionally equivalent subunits of the two adapter proteins were compared, it was observed that the B chains, that interact with clathrin heavy chain directly, showed identical clustering pattern (as shown in the Figure 6) while the subunits in close proximity with the cargo showed entirely different clustering (as shown in the Figure 7).

2] Between the two A chains of the adaptors it was noted that the sequence of the A chain of the AP1 is largely conserved across eukaryotes while that of AP2 much diverged. This difference can be attributed to the differences in the modes of biological actions of the two complexes. AP1 largely operates between golgi complex to endosomes while AP2 operates at plasma membrane. Thus, it can be imagined that AP2 caters to larger variety of cargo and hence, to a larger variety of accessory proteins compared to AP1.

**Figure 8:**
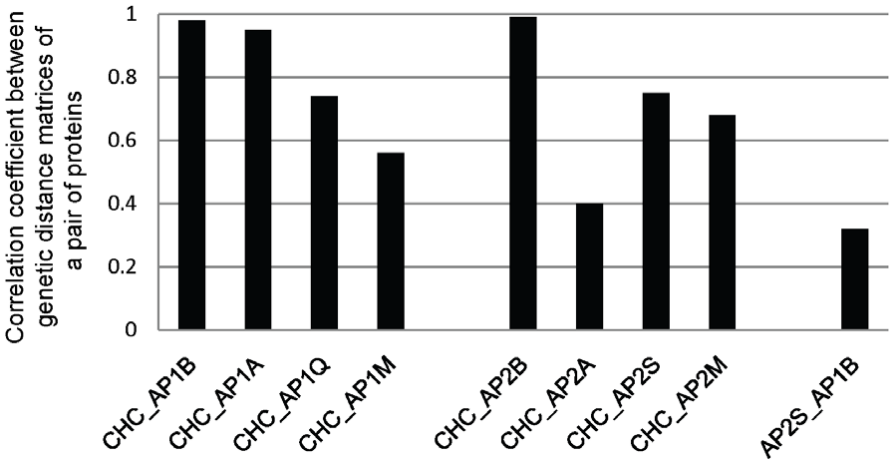
Correlation between genetic distance matrices of a pair of protein families. To investigate the correlated evolution of the adaptor protein chains with clathrin heavy chain the genetic distance matrices were generated for the orthologous sequences of every chain and compared to that of clathrin heavy chain. The comparison of the two matrices was expressed as Pearson correlation coefficient value computed. The figure summarizes the comparison of the Pearson correlation coefficients obtained for all the subunits of adaptor proteins when compared with clathrin heavy chain (as listed on X-axis).

To investigate the possibility of correlated evolution between the subunits of adaptins and clathrin heavy chain, genetic distance matrices were constructed using TREE-PUZZLE. Comparison was carried out between the matrices of adaptor protein subunits and that of clathrin heavy chain and Pearson correlation coefficients were computed. As shown in the Figure 8, maximum correlation was observed between the clathrin heavy chain and B subunits of both AP1 and AP2. Least correlation was observed between clathrin heavy chain and the M subunits of AP1 and AP2. To estimate the correlation arising merely due to speciation, the distance matrices of the two unrelated subunits from two adaptins were compared and correlation coefficient was computed, as shown in the plot in the Figure 8.

**Figure 9:**
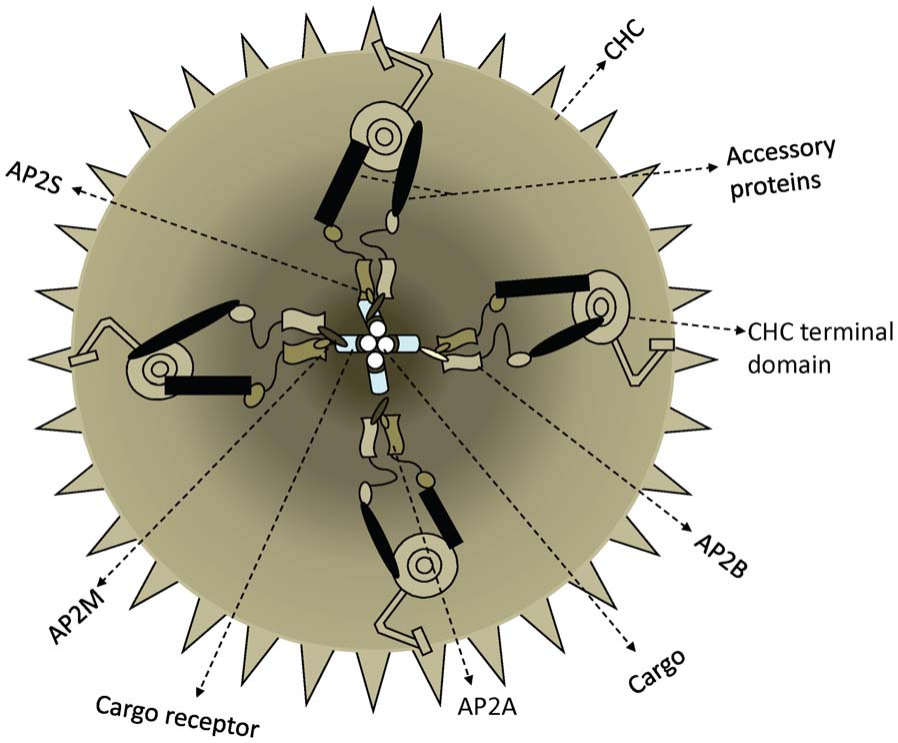
Distribution of evolutionary constraints in the clathrin coated vesicle assembly in the form of a cartoon. If clathrin coated assembly can be imagined as a sphere, with cargo being at the center while clathrin heavy chain were being at the periphery, then the figure provides view of this assembly as a transverse section of this sphere. Different components (the subunits of the complexes) of the assembly are labeled appropriately in the figure. The shaded background depicts the observed pattern in evolutionary constraints, dark depicting maximum variation in sequence (least constraint), as observed towards centre of the assembly, while the lighter shades indicate less sequence divergence (maximum constraint) as seen more towards the periphery.

Thus, if different components of the Clathrin coated assembly can be imagined to be arranged in spherical fashion with clathrin heavy chain being at the periphery and the cargo molecules at the center of the sphere, as depicted in the Figure 9, we observed radial distribution of evolutionary constraints, maximum being at the periphery and minimum being towards center.

## Discussion

The structures of clathrin coat with and without auxilin peptide bound to clathrin heavy chain are the only available structures that represent the intact clathrin coated vesicle assembly the best. However, these structures were solved at very low resolution and provide Cα atom positions only. Hence, deriving in-depth knowledge about the residues participating in protein-protein interactions had been a difficult task. Recently, we have developed a method which can perform the above mentioned task with high accuracy and sensitivity [28] and which was successfully applied to predict protein-protein interaction interfaces in dengue virus coat proteins [29] from the low resolution cryo-EM structures of virus particles. In the present analysis we have applied our method to dissect out functionally important residues on clathrin chain as well as adaptor proteins, the two busiest hubs in the interaction network presenting clathrin coated vesicles. The findings of the analysis indicated that the heavy chains of clathrin in a vesicle do not interact among themselves through identical residues, suggesting an asymmetric arrangement of the interacting partners. The possibility of this observation being an experimental artifact cannot be ruled out. The interactions amongst the heavy chains of clathrin appeared to be taking place through the CHC repeats in clathrin leg. Comparative analysis of the interacting interfaces of clathrin cage in absence and in presence of auxilin revealed that upon binding to the accessory protein peptide the interfaces on heavy chains shift but remain restricted within the repeat sequences. Due to this, although the interacting residues in the two cases changed, the residue types and thus, the interaction types were found to be largely conserved. Thus, having tandem repeats in proteins such as clathrins might be a way of providing the flexibility to accommodate varied volumes in the cage yet conserving the protein-protein interactions that provide strength to the lattice. In a few instances the clathrin heavy chain get phosphorylated at Y1477 and Y1487, which is implicated in actin remodeling and movement of the clathrin vesicle in cell [48]. Although the residues are not directly involved in any interactions reported in the present analysis, they are very close to the interface.

Adaptor proteins interact with almost every member of the vesicle and the tasks are very well shared by all the four subunits of the adaptin complex. Every subunit comprises two distinct interacting interfaces namely the one for interactions within the adaptin complex to form core and the other to interact with its non-adaptin interacting partner. The interfaces holding the subunits of the complex together seemed to be located largely towards the center of the polypeptide while in case of α and β subunits the appendages towards the N-termini harbored the interfaces holding the accessory proteins and clathrin heavy chain respectively. The interface residues inferred in the present analysis showed better residue conservation over their non-interface, surface exposed counterparts, thus validating our findings.

Owing to the functions performed by the assembly, the importance of the assembly to almost all the eukaryotic organisms can very well be imagined. Such assemblies will have a few commonalities such as the presence of clathrin like molecule to form cage in order to carry the proteins safely from place to place. However, due to the varying sizes and natures of the cargo there will be significant changes in the structures of the assembly. In order to understand the evolutionary trends in the components of clathrin vesicles detailed phylogenetic analysis was carried out and data was compared across the members of the assembly. In an organism, if members of the vesicular assembly can be imagined to be arranged in a sphere with the clathrin heavy chains being at the periphery while the cargo were being at the center, the adaptor proteins will occupy the space in between, connecting the two layers. This is the simplified model to visualize the arrangement of the components of clathrin coated vesicles. Here, we are not differentiating clathrin coated pits from plaques as elegantly shown by Saffarian et.al experimentally [49], [50], [51]. Our depiction of the clathrin assembly is close to the conventional representation of this assembly [3], [15], [52]. The findings of the phylogenetic analysis suggested a radial distribution of the evolutionary constraints with the maximum evolutionary pressure being at the periphery and hence, maximum conservation of the protein sequence seen in case of clathrin heavy chain. Constraints get reduced as we move closer to the centre that is nearer to the cargo. The analysis clearly revealed that the subunit of adaptor protein complex that interacts directly with clathrin heavy chain (the β2 subunit in AP2 and β1 in AP1) showed maximum correlation with clathrin heavy chain when the genetic distance matrices of the two proteins were compared. On the other hand, the subunit in close interactions with the cargo receptor (μ subunit) showed least correlation with clathrin heavy chain in a similar comparison. Interestingly, the phosphorylation site on the μ subunit, T156, which increases its receptor binding affinity [3], showed a complete conservation. Thus, it suggests a common regulatory mechanism existing for the cargo receptor binding of the adaptor protein, across eukaryotes, despite the differences in the nature of cargo. The same conservation pattern was observed in the sequence comparison of the interface residues, across eukaryotes. The observation is in fact highly intuitive. Across the eukaryotic organisms, although clathrin coated vesicles are recruited to transport cargo molecules from a location to another the nature of cargo being carried varies drastically. Thus, members of the assembly interacting with cargo are expected to show less sequence conservation. However, as the interacting interfaces on adaptor proteins are well separated, located on separate subunits, the change in cargo can well be accommodated in spite of keeping the other subunits minimally changed. The clathrin lattice provides added advantage by providing flexibility to accommodate varied cargo molecules, perhaps by their protein-protein interactions through the tandem repeat sequences.

In conclusion, an extensive and non-trivial task of interface determination from a low resolution structure of clathrin coat, followed by a systematic sequence analysis and visualizing the results in the context of 3D structure, enabled us to dissect out a complex pattern of radial distribution of evolutionary constraints. Given the low resolution structures, such an analysis can be extended to other large biomolecular assemblies in the cell that play crucial roles in various cellular pathways.
